# Secretory breast cancer in a boy: A case report with genetic analysis using next-generation sequencing and literature review

**DOI:** 10.1097/MD.0000000000034192

**Published:** 2023-07-07

**Authors:** Lili Deng, Yang Li, Jincai Zhong

**Affiliations:** a Department of Oncology, The First Affiliated Hospital of Guangxi Medical University, Nanning, Guangxi Province, PR China.

**Keywords:** breast carcinoma in children, case report, ETV6-NTRK3 fusion, genetics, male breast cancer, secretory breast cancer

## Abstract

Male secretory breast cancer is a rare, low-grade carcinoma, especially in boys. Due to its rarity, not much is known about this disease.

**Patient concerns:**

A 5-year-old boy presented with a 1.4 cm painless mass in the right breast.

**Diagnoses:**

Ultrasonography could not distinguish whether the breast tumor was benign or malignant. After a biopsy of the lumpectomy specimen, it was diagnosed to be secretory breast carcinoma.

**Interventions:**

The patient underwent a modified radical mastectomy for his right breast. No postoperative chemotherapy or radiotherapy was performed. Next-generation sequencing of 211 cancer-related genes was detected, and the results revealed an ETV6-NTRK3 translocation and a PDGFRB c.2632A > G mutation. None of the most commonly altered molecules in male aggressive breast cancer (such as BRCA1-2, TP53, RAD51C, and RAD51D mutations) has been identified.

**Outcomes:**

The patient was still free from local recurrence or metastases at 6-month follow-up.

**Lessons:**

The genomic profile of male pediatric SCB is relatively simple, no other known driver genes have been found except for the ETV6-NTRK3 fusion. Our report will improve our understanding of secretory breast cancer.

## 1. Introduction

Secretory breast cancer (SBC) is a rare kind of breast cancer, accounting for 0.15% of all invasive breast cancers, with a male-to-female ratio of 1:31 according to National Cancer Database analysis.^[1]^ Because of its rarity, little is known about this disease. Here, we describe a case of SBC in a 5-year-old boy and provide a genetic study of 211 cancer-related genes using capture-based next-generation sequencing. We also provide a summary of what is currently known about male pediatric SBC.

## 2. Case presentation

**Figure 1.**
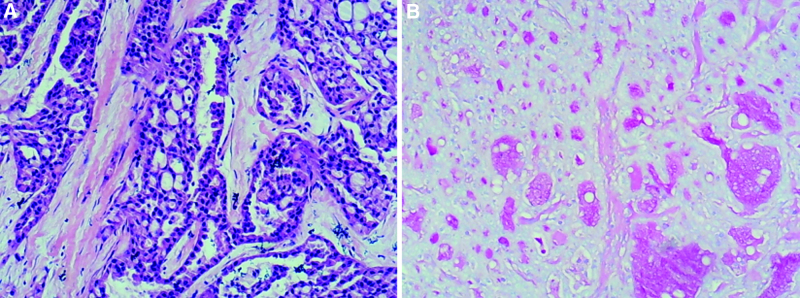
A biopsy of the lumpectomy specimen was diagnosed to be secretary breast carcinoma.

In April 2022, a 5-years old Chinese boy with no family history of relevant malignancy was admitted for a 10-month history of a right breast mass. Physical examination revealed a firm, painless, well-defined margin mass, with no clinically enlarged lymph node in the axilla or supraclavicular fossa. The boy’s growth was appropriate for his age. Ultrasonography revealed a 1.4 × 0.8 cm, well-defined hypoechoic nodule, with a few vascularizations. Blood routine, serum biochemical and tumor markers were normal. After an excisional biopsy of the breast lump, the patient was diagnosed as secretary breast carcinoma (Fig. 1). Microscopically, there was no perineural or vascular invasion.

Immunohistochemical staining of the tumor cells demonstrated negativity for estrogen receptor (ER), progesterone receptor, and human epidermal growth factor receptor 2. The Ki-67 labeling index was approximately 5%. Next-generation sequencing of 211 cancer-related genes was performed to evaluate the patient’s genetic alterations. The results are shown in Table 1. The CT examination of chest and abdomen were negative for metastases. Following the diagnosis, the patient subsequently underwent a right modified radical mastectomy. The postoperative pathological results showed the mastectomy specimen was negative for residual neoplasia and no lymph node metastasis (0/12). Because there were no distant metastases or deaths associated with male pediatric SBCs and our patient had a small tumor with no vascular involvement and no axillary lymph node metastases, he appears to have low malignant potential. Moreover, we took into account the likelihood of impaired growth following adjuvant radiation or chemotherapy therapy in a young boy, so there was no additional treatment for the patient after the surgery. Neither chemotherapy nor radiotherapy were instituted after the operation. The patient was well at 6-month follow-up.

**Table 1:** Genome analysis of a boy secretory breast carcinoma.

Gene name	Variant information	Position	Variation in abundance
ETV6-NTRK3	Gene fusion	E5N15	31.8%
PDGFRB	NM_002609.4 c.2632A > G p.S878G	exon19	1.53%

## 3. Literature review and discussion

Although SBC was originally described as “juvenile breast carcinoma,” only 15 cases^[2–15]^ have been reported in male children (under 14 years of age) (Table 2). Clinically, a slow-growing, painless breast mass is the main sign. The average tumor size of male pediatric SBC patients is 1.7 cm (range 0.8–3.5 cm), and 20% (3/15) of the reported cases demonstrated axillary lymph node metastases at initial diagnosis. By comparison, the adult patients had a larger average tumor size and a higher rate of lymph node metastases.^[1]^ The majority of these pediatric SBC patients are negative for ER, progesterone receptor, and human epidermal growth factor receptor 2, with only 6.6% (1/15) having modest expression of hormone receptors. This is different from other types of male breast cancer since >90% of the latter patients are positive for ER.^[16]^ Further, according to the Surveillance, Epidemiology, and End Results database, the positive rates of ER can reach 58% in the entire population of SBC patients, making this percentage of hormone receptor expression substantially lower in children with SBC than in adults with SBC.^[17]^

**Table 2:** Cases of male patients with SBC published in literature.

Author	Year	Age （yr）	Duration of symptoms	Size (mm)	Hormone receptors	Gene testing	Axillary status	Treatment	Family history	Follow-up (mo)
Simpson et al	1969	5	ND	ND	ND	ND	NEG	ND	ND	NED 48
Tavassoli et al	1980	9	ND	ND	ND	ND	NEG	ND	ND	NED 21
Vieni et al	1985	3	1 mo	15	ND	ND	POS（1/4）	SM + ALNS	ND	ND
Chevallier et al	1999	9	14 mo	20	ER−, PgR−	ND	NEG	LE + ALND	ND	NED 45
Yildirim et al	1999	11	1 yr	15	ER−	ND	POS (1/18)	MRM + CT + RT	ND	NED 12
Bhagwandeen et al	1999	9	1 mo	12	ER−, PgR−	ND	NEG (0/15)	MRM	ND	ND
Titus et al	2000	9	1 mo	10	ER−, PgR−	ND	NEG (0/15)	SM + ALND	ND	NED 20
Szanto et al	2004	7.5	6 mo	17	ER−, PgR−	BRCA1-2 mutations (-)	NEG	SM + SLNB	His maternal aunt had breast cancer	NED 7
Cabello et al	2011	13	4 yr	15	ER−, PgR−	ND	POS (1/28)	SM + ALND + CT +RT	ND	NED 120
Li et al	2012	10	0.5 mo	20	ER−, PgR−、HER-2-	ND	NEG(0/11）	MRM	ND	NED 12
Zhou et al	2013	10	2 yr	20	ER−, PgR−、HER-2-	ND	NEG(0/12)	MRM	ND	NED 13
Ghilli et al	2018	5.5	4 mo	8	ER−, PgR−，HER-2-	BRCA1-2 mutations (-), a 3q28 duplication: arr3q28 × 3 (GRCh37)	NEG	LE + SLNB	His paternal grandfather had colon cancer, melanoma, tubular BC. The paternal grandfather’s brother had colon cancer, the nephew had BC, the paternal grandmother’s sister had BC.	NED 48
Novochadlo et al	2020	9	4 yr	35	ER+, PgR+、HER-2-	ND	NEG	SM + ALND	ND	NED 13
Chen et al	2022	7	ND	21	ER-, PgR-、HER-2-	ETV6 rearrangement	NEG(0/4)	SM + SLNB	ND	NED 51
This case (Deng et al)	2022	5	10 mo	14	ER-, PgR-、HER-2-	ETV6-NTRK3 fusion, PDGFRB c.2632A > G mutation	NEG (0/12)	MRM	ND	NED 6

ALND = axillary lymph node dissection, ALNS = axillary lymph node sampling, CT = chemotherapy, ER = estrogen receptor, HER-2 = human epidermal growth factor receptor 2, LE = local excision, MRM = modified radical mastectomy, ND = not defined, NED = no evidence of disease, NEG = negative, PgR = progesterone receptor, POS = positive, RT = radiotherapy, SBC = secretory breast cancer, SLNB = sentinel lymph node biopsy, SM = simple mastectomy.

Among those male pediatric SBC, there were 2 patients^[9,13]^ (13%, 2/15)had a family history of malignant tumors, but no BRCA1-2 mutations mutations were found in these individuals. To present, only few genome sequencing studies in male pediatric SCB have been completed (Table 2), and the genetic characteristics reflecting the biological behaviors of this disease are still poorly known. Our case’s genetic research revealed a nonrecurrent PDGFRB genetic variation and an ETV6-NTRK3 fusion. In our case, none of the most frequently changed molecules in aggressive male breast cancer (such BRCA1-2, TP53, RAD51C, or RAD51D mutations) were found. According to the literature and the results of our genome sequencing, the genomic profile of male SCB is quite straightforward; the only oncogenic driver identified so far is the ETV6-NTRK3 fusion. A 3q28 duplication was also once discovered in a male pediatric SCB with a family history of a pertinent cancer using array-CGH analysis and Real-time PCR, but the author did not think that this duplication was responsible for the patient’s condition.^[13]^ PDGFRB is a classical proto-oncogene, that encodes receptor tyrosine kinases responding to platelet-derived growth factor. PDGFRB mutations have been identified in gastric cancer, hematopoietic, glial and soft-tissue cancers, which may play a role in cancer development.^[18]^ However, there is no evidence to prove that the PDGFRB c.2632A > G mutation could play a part in the pathogenesis of any tumor diseases based on the current literature. ETV6-NTRK3 fusion is characteristic of SBC, >90% of SBC harbor this fusion, but it has not been identified in other types of breast cancer.^[19]^ The ETV6-NTRK3 fusion protein causes constitutive activation of the tropomyosin receptor kinase C protein through activating the ras-mitogen-activated protein kinase mitogenic pathway as well as the phosphatidylinositol 3-kinase pathway, which ultimately leads to cellular transformation and oncogenesis.^[20]^ NTRK inhibitors have been shown in recent studies to reduce clinically aggressive SBC instances.^[14,21]^ In the event of distant metastasis, NTRK inhibitors would be a preferred therapy choice for ETV6-NTRK3 fusion SBC patients.

Even when there were axillary lymph node metastases, the SBC was regarded as a low-grade malignant tumor with a better result than other kinds of breast cancer. So far, no metastases have been documented in the series of male pediatric patients. At present, there are no standard treatment guidelines for SBC. For pediatric SBC, surgery is the primary treatment, but the extent of surgery and whether to choose subsequent adjuvant therapy are still controversial. According to the research, local excision/simple mastectomy combined with sentinel lymph node biopsy/axillary lymph node dissection was used to treat 61.5% (8/13) of male pediatric SBC patients and modified radical mastectomy was used to treat 38% (5/13) of the patients. Male pediatric patients looked to benefit from both local excision and simple mastectomy as treatments, but many doctors now favor the latter procedure to lower the chance of local recurrence.^[22]^ Given that the ratio of lymph node metastasis was higher in male patients than in the general population, and that lymph node metastasis may occur more frequently in smaller tumors in male patients, sentinel lymph node biopsy or axillary lymph node dissection is required in male pediatric SBC patients.^[22,23]^ Despite the fact that local radiotherapy and postoperative chemotherapy were administered to 66.6% (2/3) of patients with axillary lymph node metastases, current thinking contends that pediatric SBC should not be advised to receive postoperative adjuvant radiotherapy. Because some research suggests that radiation exposure in youngsters can harm the heart and lungs, hinder growth, and is not good for long-term survival.^[17,24]^ Adjuvant chemotherapy has been advocated by some researchers for patients with lymph node-positive tumors, but others have argued against using adjuvant chemotherapy for pediatric SBC due to the minimal risk of metastasis.^[9,25]^

## 4. Conclusion

Male SBC is a very rare disease. Here we describe a case of male SBC in a 5-year-old kid with a genetic study and review the literature for investigations of male pediatric SBC. Male SBC is a very rare condition. With the exception of the ETV6-NTRK3 fusion, the genomic profile of male pediatric SCB is rather straightforward and lacks any recognized driver genes. In our patient, none of the most frequently changed molecules in aggressive male breast cancer (including BRCA1-2, TP53, RAD51C, or RAD51D mutations) were found. The most vital form of therapy is surgical resection. Adjuvant postoperative radiation is not advised for pediatric SBC. The use of adjuvant chemotherapy in pediatric male SBC is not sufficiently supported by the available research.

## Author contributions

**Conceptualization:** Lili Deng.

**Supervision:** Jincai Zhong.

**Writing – original draft:** Lili Deng.

**Writing – review & editing:** Jincai Zhong, Yang Li.
